# Enterovirus type 71 2A protease functions as a transcriptional activator in yeast

**DOI:** 10.1186/1423-0127-17-65

**Published:** 2010-08-04

**Authors:** Chee-Hing Yang, Hui-Chun Li, Jeng-Geng Jiang, Che-Fang Hsu, Yi-Jen Wang, Meng-Jiun Lai, Yue-Li Juang, Shih-Yen Lo

**Affiliations:** 1 Department of Laboratory Medicine and Biotechnology, Tzu Chi University, Hualien, Taiwan; 2 Department of Biochemistry, School of Medicine, Tzu Chi University, Hualien, Taiwan; 3 Graduate Institute of Medical Biotechnology, Tzu Chi University, Hualien, Taiwan; 4 Department of Microbiology, School of Medicine, Tzu Chi University, Hualien, Taiwan; 5 Department of Laboratory Medicine, Buddhist Tzu Chi General Hospital, Hualien, Taiwan

## Abstract

Enterovirus type 71 (EV71) 2A protease exhibited strong transcriptional activity in yeast cells. The transcriptional activity of 2A protease was independent of its protease activity. EV71 2A protease retained its transcriptional activity after truncation of 40 amino acids at the N-terminus but lost this activity after truncation of 60 amino acids at the N-terminus or deletion of 20 amino acids at the C-terminus. Thus, the acidic domain at the C-terminus of this protein is essential for its transcriptional activity. Indeed, deletion of amino acids from 146 to 149 (EAME) in this acidic domain lost the transcriptional activity of EV71 2A protein though still retained its protease activity. EV71 2A protease was detected both in the cytoplasm and nucleus using confocal microscopy analysis. Coxsackie virus B3 2A protease also exhibited transcriptional activity in yeast cells. As expected, an acidic domain in the C-terminus of Coxsackie virus B3 2A protease was also identified. Truncation of this acidic domain resulted in the loss of transcriptional activity. Interestingly, this acidic region of poliovirus 2A protease is critical for viral RNA replication. The transcriptional activity of the EV71 or Coxsackie virus B3 2A protease should play a role in viral replication and/or pathogenesis.

## Background

Enterovirus type 71 (EV71) is the causative agent of several human diseases, including hand-foot-and-mouth disease, encephalitis, and meningitis. EV71 is a single-stranded, positive-sense RNA virus, which belongs to the *Picornaviridae *family [1]. Genomic RNA of picornaviruses (e.g. polioviruses) encodes a polyprotein precursor, which is processed by three proteases (the maturation protease, 2A protease, and the 3C protease) into at least 11 different proteins, which are arranged in the order of NH2-VP4-VP2-VP3-VP1-2A-2B-2C-3A-VPg-3C-3D-COOH [1]. The 2A protease of poliovirus, a representative member of the *Picornaviridae*, is a cysteine protease with multiple functions [2]. Similar to poliovirus 2A protease, expression of EV71 2A protease led to cleavage of the eukaryotic initiation factor 4GI, a key factor for host protein synthesis [3,4]. Moreover, transient expression of EV71 2A protease alone also resulted in the induction of apoptotic change [5,6]. However, the function of EV71 2A protease is not well characterized. The biologic function of EV71 2A protease was investigated by fusing it with the DNA-binding domain of Gal4 and examining its possible interaction with cellular factors [7].

## Materials and Methods

### Plasmid construction

Procedures used in our previous studies were followed to construct the plasmids [8,9]. The PCR primers used in this study are listed in Table 1. To clone the DNA fragment encoding the full-length EV71 2A protease (nucleotides from 3332 to 3781 of strain pinf7-54A) for yeast two-hybrid screening, oligonucleotide primers (2AY-S and 2AY-AS) were used to perform PCR. After the PCR, the DNA fragment was treated with T4 polynucleotide kinase, digested by the restriction enzyme EcoRI, and cloned into the pBDGal4 Cam (Stratagene, USA) expression vector, which had been linearized with EcoRI and SmaI. Using the same approach, PCR was performed with primer pairs (2AY-21 S and 2AY-AS, 2AY-41 S and 2AY-AS, 2AY-61 S and 2AY-AS) to clone the DNA fragments encoding EV71 2A protease with the N-terminal truncation of 20, 40, 60 amino acids respectively, while another PCR was performed with primer pairs (2AY-S and 2AY-130AS, 2AY-S and 2AY-110AS, 2AY-S and 2AY-90AS) to clone the DNA fragments encoding EV71 2A protease with the C-terminal deletion of 20, 40, 60 amino acids respectively. Primers (2AY-S and 2AY-AS101) were used to perform PCR to clone the DNA fragment encoding EV71 2A protease without amino acids from 146 to 149 using the same approach.

**Table 1:** PCR primers used in this study

Name	Sequence
2AY-S	(5'-G*GAATTC*GGGAAATTTGGACAG-3')
2AY-AS	(5'-CCG*CTCGAG***TTA**CTGCTCCATGGCTTC-3')
2AY-21S	(5'-G*GAATTC*CATCTTGCTACTCATAA-3')
2AY-41S	(5'-G*GAATTC*CTCGTATCATCTACCAC-3')
2AY-61S	(5'-G*GAATTC*GGAGTGTATTATTGTAA-3')
2AY-90AS	(5'-**TTATTA**ATAATACTCGCTGGCCTC-3')
2AY-110AS	(5'-**TTATTA**GCAATCCCCTGGTTCCGA-3')
2AY-130AS	(5'-**TTATTA**GCAATCCCCTGGTTCCGA-3')
VP1/2A-S	(5'-CC*ATCGAT***ATG**ATGGGTACGTTC-3')
2A-S10	(5'-G*GAATTC***ATG**GGGAAATTTGGACAGCAG-3')
2A-AS2	(5'-GC*TCTAGA***CTA**CTGCTCCATGGCTTCATCATC-3')
2A-AS3	(5'-GC*TCTAGA*CTGCTCCATGGCTTCATCATC-3')
C110A-S	(5'-CCAGGGGAT***GCC***GGTGGCATTCTTAGATGC-3')
C110A-AS	(5'-AATGCCACC***GGC***ATCCCCTGGTTCCGAATG-3')
L30/43-S	(5'-CATAATGACTGGGCAAACTCATCTACCACTGCTCAA-3')
L30/43-AS	(5'-TTGAGCAGTGGTAGATGAGTTTGCCCAGTCATTATG-3')
2AY-AS101	(5'-CCGCTCGAG**TTA**CTGATCATCCAACCACAGAAG-3')
2A-AS301	(5'-GC*TCTAGA*CTGATCATCCAACCACAGAAG-3')
CoxB2AY-S	(5'-G*GAATTC***ATG**GGACAACAATCAGGGGC-3')
CoxB2AY-AS	(5'-**TTATTA**CTGTTCCATTGCATCATC-3')
CoxB2AY-61S	(5'-G*GAATTC*TTTTGTGCGTCCAAAAAC-3')
CoxB2AY-127AS	(5'-**TTATTA**GCCTTCACCCCCCATGGT-3')
PCBP2-S	(5'-CTCTCACCATCCGGCTACTTAT-3')
PCBP2-AS	(5'-GCTGCTTATGTCCTCTTCCAGT-3')
PTBP1-S	(5'-CTACATCCAGTTCTCCAACCAC-3')
PTBP1-AS	(5'-GCTGCTTATGTCCTCTTCCAGT-3')
RTN3-S	(5'-ACTCTGTCCTCAGAAGCTTTCC-3')
RTN3-AS	(5'-CTCATAGACAATCGGGACACTG-3')
GBF1-S	(5'-CCCACTATTGCTGCTCTCTCTT-3')
GBF1-AS	(5'-CTGGGCAGGTTCTCAATAGACT-3')
CD55-S	(5'-CCGTCTTCTATCTGGTTCTCGT-3')
CD55-AS	(5'-GTTACTAGCGTCCCAAGCAAAC-3')
SAM68-S	(5'-CGAAGGCTATTACAGCCAGAGT-3')
SAM68-AS	(5'-CATATGGGTGCTCTCTGTATGC-3')

Note: Nucleotides for restriction enzyme cutting sites are italicized. Nucleotides for point mutations are bold and italicized. Nucleotides for start and stop codons are marked with bold letters. Primers for the detection of cellular genes were used in real-time RT-PCR.

To clone the DNA fragment encoding the full-length Coxsackie virus B3 2A protease for yeast two-hybrid screening, mRNA extracted from a patient infected with Coxsackie virus B3 was converted into cDNA and oligonucleotide primers (CoxB2AY-S and CoxB2AY-AS) were used to perform PCR (the sequence is the same as nucleotides from 3304 to 3744 of GI:323419). PCR was performed using primer pairs (CoxB2AY-61 S and CoxB2AY-AS) to clone the DNA fragments encoding Coxsackie virus 2A protease with the N-terminal truncation of 60 amino acids, while another PCR was performed with primer pairs (CoxB2AY-S and CoxB2AY-127AS) to clone the DNA fragments encoding Coxsackie virus 2A protease with the C-terminal deletion of 20 amino acids. Again, after the PCR, the DNA fragments were treated with T4 polynucleotide kinase, digested by the restriction enzyme EcoRI, and cloned into the pBDGal4 Cam (Stratagene, USA) expression vector which had been linearized with EcoRI and SmaI.

To clone the DNA fragment encoding the C-terminus of EV71 VP1 and the full-length 2A protease (nucleotides from 3124 to 3781 of strain pinf7-54A) for transient expression in mammalian cells, PCR was performed using oligonucleotide primers (VP1/2A-S and 2AY-AS2). After the PCR, the DNA fragment was digested by restriction enzymes (ClaI/XbaI), together with the EMCV IRES sequence (digested with EcoRI/ClaI), and cloned into the expression vector pcDNA3 (Invitrogen, USA) which had been linearized with EcoRI/XbaI. To mutate amino acid 110 of EV71 2A protease from Cys to Ala, primers (VP1/2A-S and C110A-AS) were used to amplify the 5'-end of the gene fragment while primers (C110A-S and 2AY-AS2) were used to amplify the 3'-end fragment. These two DNA fragments were linked together by PCR using primers (VP1/2A-S and 2AY-AS2). After the PCR, the DNA fragment was digested by restriction enzymes (ClaI/XbaI), together with the EMCV IRES sequence (digested with EcoRI/ClaI), and cloned into the expression vector pcDNA3 (Invitrogen, USA) which had been linearized with EcoRI/XbaI.

To clone the DNA fragment encoding the C-terminus of EV71 VP1 and full-length 2A protease with the V5 tag in the C-terminus for confocal microscopy analysis in mammalian cells, PCR was performed using oligonucleotide primers (VP1/2A-S and 2AY-AS3). After the PCR, the DNA fragment was digested by restriction enzymes (ClaI/XbaI), together with the EMCV IRES DNA sequence (digested with EcoRI/ClaI), and cloned into the expression vector pcDNA3.1-V5-His A (Invitrogen, USA) which had been linearized with EcoRI/XbaI. To clone the EV71 2A protease with mutation of amino acid 110 from Cys to Ala for confocal microscopy analysis, the DNA template containing this mutation and primers (2A-S10 and 2A-AS3) was used to amplify the DNA fragment of full-length EV71 2A protein with mutation of amino acid 110 from Cys to Ala. After the PCR, the DNA fragment was digested by the restriction enzymes (EcoRI/XbaI), and cloned into the expression vector pcDNA3.1-V5-His A (Invitrogen, USA) which had been linearized with EcoRI/XbaI. To clone the EV71 2A protease without potential NES (amino acid 31 to 42) for confocal microscopy analysis, the DNA fragment containing the mutation of amino acid 110 from Cys to Ala was used as the PCR template. Primers (2A-S10 and L30/43-AS) were used to amplify the 5'-end of the gene fragment while primers (L30/43-S and 2A-AS3) were used to amplify the 3'-end fragment. These two DNA fragments were linked together by PCR using primers (2A-S10 and 2A-AS3). After the PCR, the DNA fragment was digested by restriction enzymes (EcoRI/XbaI), and cloned into the expression vector pcDNA3.1-V5-His A (Invitrogen, USA) which had been linearized with EcoRI/XbaI. The same approach was used to clone the DNA fragment encoding the C-terminus of EV71 VP1 and 2A protease deleting the amino acids 146-149 with the V5 tag in the C-terminus using primers (VP1/2A-S and 2A-AS301) to perform PCR.

All of the expression plasmids were verified by sequencing.

### Yeast two-hybrid screening

The yeast two-hybrid system used for screening was purchased from Clontech Laboratories (USA). The experimental procedures were conducted according to the manufacturer's instructions.

### Protein expression and Western blot analysis

HeLa cells were maintained in RPMI (Chemicom, USA) medium containing 10% fetal bovine serum, 1% glutamine (200 mM, Gibco, USA), and 100 ug/ml penicillin/streptomycin (Gibco BRL, USA). Cultured cells were maintained at 37°C with 5% CO_2_. Cells were seeded at a density of approximately 4 × 10^5 ^cells per 60-mm culture dish. After overnight incubation, cells were transfected with plasmids (1-4 ug) using the ExGen 500 *in vitro *transfection reagent (Fermentas, USA) or Arrest-In™transfection reagent (Open Biosystems, USA). At 48 hours after transfection, recombinant proteins expressed in cells were analyzed by Western blot.

Our previous procedures were followed for Western blot analysis [7,10]. Rabbit polyclonal antibodies against ERK-2 and eIF4G were purchased from Santa Cruz Biotechnology (USA). Monoclonal antibodies against PARP were purchased from SEROTEC (UK). Monoclonal antibodies against V5 tag were purchased from Invitrogen (USA). Rabbit antibodies against EV71 2A protease were generated in the lab.

### Confocal microscopy analysis

HeLa cells were seeded at a density of about 2.5 × 10^5 ^cells per 35 mm culture dish. After overnight incubation, cells were transfected with plasmids (0.5 - 2 ug) using the ExGen 500 *in vitro *transfection reagent (Fermentas, USA) or Arrest-In™transfection reagent (Open Biosystems, USA). At 48 hours after transfection, recombinant proteins expressed in cells were analyzed by confocal microscopy.

Cells with recombinant proteins were fixed with 1% methanol/acetone at 0°C for 10 minutes, washed with incubation buffer (0.05% NaN_3_, 0.02% saponin, 1% skim milk in PBS) twice for 2 minutes each, and then incubated with the anti-V5 antibody (1:200 dilution) at 37°C for 30 minutes. Cells were washed with PBS at room temperature for five minutes three times, and then incubated with Cy3-conjugated goat anti-mouse IgG antibody (1:20 dilution) at 37°C for 30 minutes. Cells were washed three more times with PBS. DAPI (Merck, Germany) was used to stain the nucleus.

### Real-time reverse transcriptase-polymerase chain reaction (RT-PCR)

HeLa cells were transfected with plasmids of vector alone or pcDNA3.1-IRES-2A using Arrest-In™transfection reagent (Open Biosystems, USA). At 24 hours after transfection, G418 was used to select the cells with transfected plasmid. After 72 hours, cellular mRNAs were extracted and our previous procedures were followed for real-time RT-PCR [11].

## Results

### EV71 2A protease exhibited strong transcriptional activity in yeast cells

**Figure 1:**
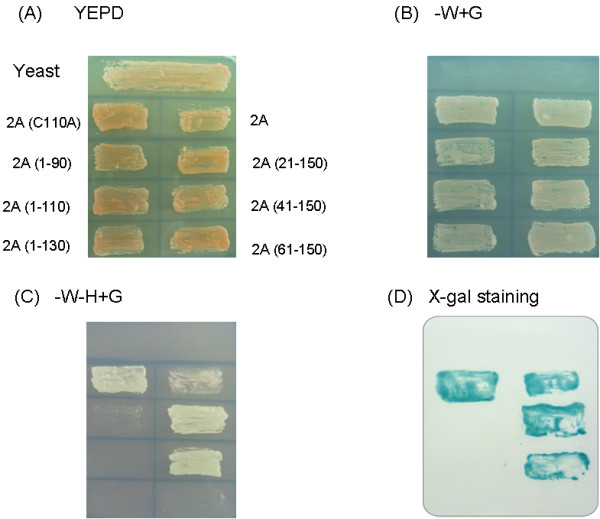
**Growth of yeasts either mock-transfected or transfected with plasmids encoding EV71 2A protease of different sizes in YEPD medium (A), YEPD without tryptophan (B), or YEPD without tryptophan and histidine (C)**. (D) X-gal staining of yeasts in (C).

EV71 2A protease, when fused with the DNA-binding domain of Gal4, activates the reporter genes in yeast cells (Figure 1). This reaction is quite specific since none of the other proteins we studied at the same time exhibited this activity, including EV 71 3C protein, hepatitis C virus NS5A protein, NS3 protein(data not shown), or ARFP [7]. Truncation of 40 but not 60 amino acids at the N-terminus of EV71 2A protease did not affect its transcriptional activation activity (Figure 1). On the other hand, deletion of 20 amino acids at the C-terminus of EV71 2A protease resulted in the loss of transcriptional activity (Figure 1).

### Transcriptional activity of EV71 2A protease is independent of its protease activity

**Figure 2:**
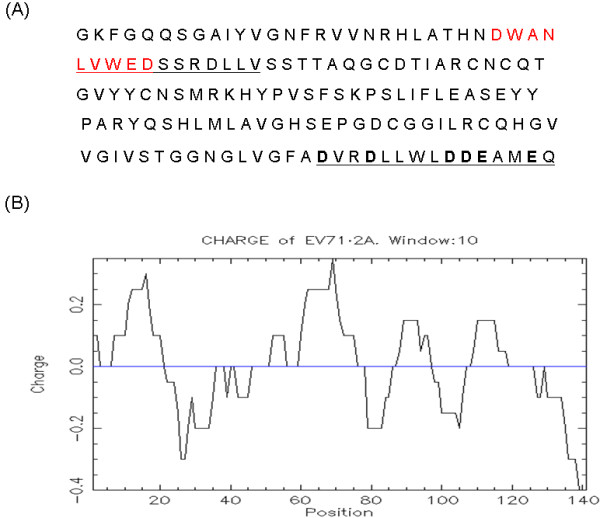
**Analysis of EV71 2A protease protein**. (A) Amino acid sequence of EV71 2A protein. The predicted 9aa TAD (a.a. 27-35) is indicated with red letters. Potential NES (a.a. 31-42) is underlined. The acidic domain (the last fifteen amino acids) is also underlined. (B) Charge distribution of EV71 2A protease: the C-terminus of this protein is highly acidic.

**Figure 3:**
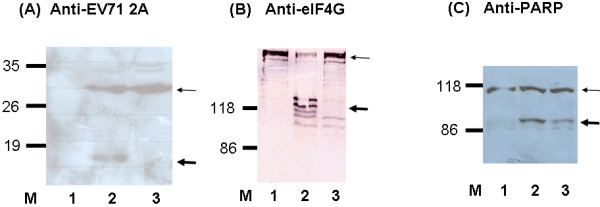
**Western blotting analysis of wild-type EV71 2A protease or with amino acid 110 mutation from Cys to Ala in HeLa cells**. HeLa cells were transfected with vector only (lane 1), or with the plasmid encoding the C-terminus of VP1 and wild-type 2A (lane 2), or with the plasmid encoding the C-terminus of VP1 and 2A with amino acid 110 mutation from Cys to Ala (lane 3). After transfection, cell lysates were analyzed and detected using rabbit anti-EV71 2A protein polyclonal antibody (A), mouse anti-eIF4G monoclonal antibody (B), or mouse anti-PARP monoclonal antibody (C). The thin arrows indicate the uncleaved proteins (VP1-2A, intact eIF4G, or intact PARP) while the thick arrows indicate the cleaved products (2A, cleaved eIF4G, or cleaved PARP).

Amino acid residues His 20, Asp 38, and Cys 109 comprise the catalytic core of poliovirus 2A protease [12]. The corresponding residue of EV71 2A protease essential for its protease activity is Cys in amino acid 110 (Figure 2A). The expression plasmids encoding the C-terminus of VP1 protein, full-length 2A protease wild-type or with mutation in amino acid 110 from Cys to Ala were constructed and transfected into HeLa cells. Mutation of amino acid 110 from Cys to Ala of EV71 2A protein blocked the auto-protease activity of this protein (Figure 3A), suppressed the cleavage of cellular eIF4G protein (Figure 3B), and reduced the induction of apoptosis in HeLa cells (Figure 3C). However, EV71 2A protease with this mutation still possessed transcriptional activity in yeast cells (Figure 1).

### Sub-cellular localization of EV71 2A protease

**Figure 4:**
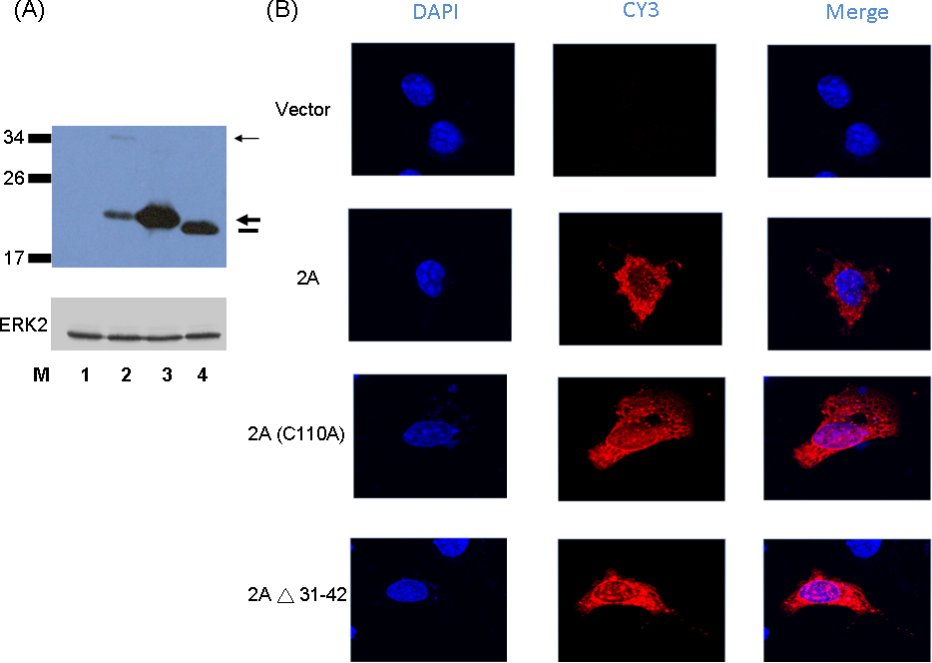
**Analysis of various EV71 2A protein mutants in HeLa cells**. (A) Protein expression of various EV71 2A protein mutants with V5 tag in the C-terminus. HeLa cells were transfected with vector only (lane 1) or with the plasmid encoding the C-terminus of VP1 and wild-type 2A (lane 2), or with the plasmid encoding 2A with amino acid 110 mutation from Cys to Ala (lane 3), or with the plasmid encoding 2A protein deleting amino acids from 32 to 41 (lane 4). After transfection, cell lysates were analyzed by Western blot using mouse anti-V5 tag monoclonal antibody. The thin arrow indicates the uncleaved protein (VP1-2A in lane 2) while the thick arrow indicates the 2A protein (lanes 2 and 3). The thick line indicates the location of 2A protein deleting amino acids from 32 to 41 (lane 4). Erk2 protein served as a loading control. (B) Confocal microscopy analysis of various EV71 2A protein mutants. After HeLa cells were transfected with the indicated plasmids, cells were fixed and stained with mouse anti-V5 tag monoclonal antibody, followed by Cy3-conjugated anti-mouse IgG. DAPI (Merck, Germany) was used to stain DNA for localization of the nucleus.

No potential nuclear localization signal (NLS) was found within the EV71 2A protease http://tw.expasy.org/index.html. However, it is known that ions, smaller metabolites, and globular proteins up to 20-40 kDa can passively diffuse through the central aqueous region of the nuclear pore complex [13]. Thus, EV71 2A protease with 150 amino acids could passively disuse into the nucleus. Confocal microscopy analysis was used to examine the sub-cellular localization of EV71 2A protein. The expression plasmid encoding the C-terminus of VP1 protein, full-length 2A protease and V5 tag was constructed and transfected into HeLa cells before confocal microscopy analysis. The same approach was used to construct and transfect the DNA fragment encoding full-length 2A protease with mutation of amino acid 110 from Cys to Ala. Protein expression of these constructs was demonstrated using Western blot analysis (Figure 4A). Both the wild-type and mutant EV71 2A proteins localized in both cytoplasm and nucleus (Figure 4B). Amino acids 31 to 42 of EV71 2A protein (Figure 2A) were identified as a potential nuclear export signal (NES) http://tw.expasy.org/index.html. However, similar to full-length EV71 2A protease, this protein without amino acids 31 to 42 localized in both the cytoplasm and the nucleus but not in the nucleus only (Figure 4B).

### Deletion of amino acids from 146 to 149 of EV71 2A protease lost its transcriptional activity but retained its protease activity

**Figure 5:**
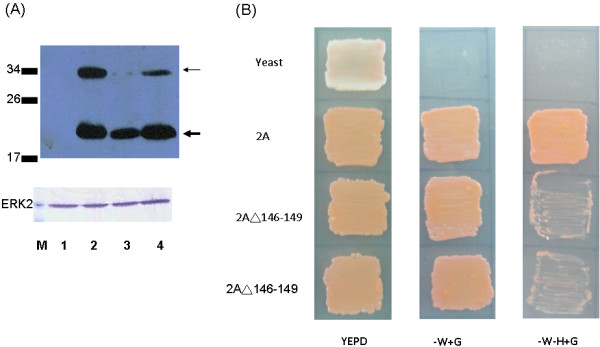
**EV71 2A protease without amino acids 146-149 still retained its protease activity but lost its transcriptional activity**. (A) HeLa cells were transfected with vector only (lane 1) or with the plasmid encoding the C-terminus of VP1 and wild-type 2A (lane 2), or with the plasmid encoding the C-terminus of VP1 and 2A deleting amino acids 146-149 (lanes 3 and 4). After transfection, cell lysates were analyzed by Western blot using mouse anti-V5 tag monoclonal antibody. The thin arrow indicates the uncleaved protein (VP1-2A in lanes 2-4) while the thick arrow indicates the 2A protein (lanes 2-4). Erk2 protein served as a loading control. (B) Growth of yeasts either mock-transfected or transfected with plasmids encoding EV71 2A or 2A protein without amino acids 146-149 in YEPD medium, YEPD without tryptophan, or YEPD without tryptophan and histidine.

A previous report demonstrated that the C-terminal acidic region of poliovirus 2Apro is critical for viral RNA replication but not for cis- or trans- proteolytic cleavage [14]. To determine whether mutation of the amino acids in the C-terminal acidic region affect its transcriptional activity, EV71 2A protease without amino acids 146-149 (EAME) was constructed. Indeed, EV71 2A protease without amino acids 146-149 still retained its protease activity (Figure 5A) but lost its transcriptional activity (Figure 5B).

### EV71 2A protease did not transactivate cellular genes reported to enhance the replication of poliovirus or EV 71

Some cellular genes were reported previously to enhance the replication of poliovirus or EV71: poly(rC) binding proteins [15-17], cellular COPII proteins [18], the polypyrimidine tract binding proteins [19], Reticulon 3 [20], and GBF1 [21]. Real-time RT-PCR was performed to determine whether EV71 2A protease could transactivate PCBP2, PTBP1, RTN3, GBF1, CD55, or SAM68 gene. However, EV71 2A protease repressed rather than transactivated all of these cellular genes (data not shown).

### Coxsackie virus B3 2A protease exhibited transcriptional activity in yeast cells

**Figure 6:**
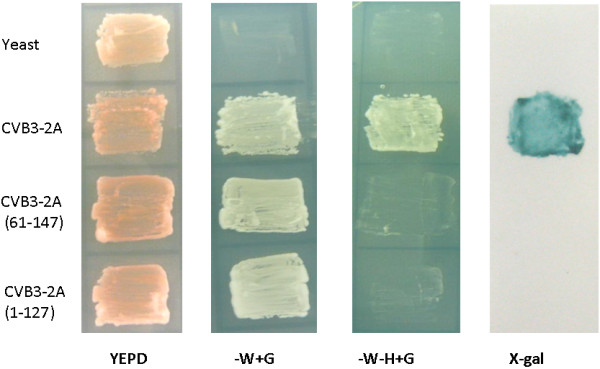
**Growth of yeasts either mock-transfected or transfected with plasmids encoding CoxB3 2A protease of different sizes in YEPD medium, YEPD without tryptophan, or YEPD without tryptophan and histidine**. X-gal staining of yeasts in YEPD without tryptophan and histidine.

To investigate whether other picornaviral 2A proteases possess transcriptional activity, the DNA fragment encoding the full-length Coxsackie virus B3 2A protease was amplified by PCR and fused with the DNA-binding domain of Gal4. This fusion protein also activates reporter genes in yeast (Figure 6). Again, Coxsackie virus B3 2A protease lost its transcriptional activity after truncation of 60 amino acids at the N-terminus or deletion of 20 amino acids at the C-terminus (Figure 6).

## Discussion

EV71 2A protease is expected to enter the nucleus by passive diffusion since it is a small protein with no potential NLS. This protein would not be actively exported from the nucleus since no functional NES was detected (Figure 4). These findings explain why only small portion of EV71 2A protease localized in the nucleus and the majority of this protein was retained in the cytoplasm (Figure 4). Interestingly, 2A proteins of poliovirus and EMCV were reported to localize in the nucleus [22,23].

As a transcriptional activator, EV71 2A protease did not contain a glutamine-rich domain, a leucine zipper domain, or a proline-rich domain as are found in some other eukaryotic transcriptional activators such as CTF/NF-1 or the amino terminal deletion mutants of HCV NS5A protein [24-27]. The PXXXP motif necessary for full transactivation of HIV Tat protein was also not found in EV71 2A protease (Figure 2A) [28]. However, one acidic domain (rich in Glu (E) or Asp (D), Figure 2B), functioning universally in eukaryotic transcriptional activators from yeast to human [29,30], was found in the C-terminus of EV71 2A protease (6 amino acids within the last 15 amino acids are acidic, Figure 2A). Moreover, 9aa TAD possessing an autonomous transactivation activity in yeast and mammalian cells was also found at the N-terminus of EV71 2A protease (from aa 27 to 35) (Figure 2A) [31]. Deletion analysis revealed the acidic domain in the C-terminus but not 9 aa TAD in the N-terminus of EV71 2A protease is essential for the transcriptional activation activity of this protein (Figure 1).

In addition to EV71 2A protease (Figure 1), Coxsackie virus B3 2A protease is also a transcription activator (Figure 6). Interestingly, there is an acidic domain in the C-terminus of this protein (6 amino acids within the last 15 amino acids are acidic, Table 2). The 2A proteases of other members of the *Enterovirus *genus, such as Coxsackie viruses and polioviruses, also contain an acidic domain in the C-terminus (Table 2). On the other hand, there is no such an acidic domain in the C-terminus of 2A proteases of rhinoviruses (2 or 3 amino acids within the last 15 amino acids are acidic, Table 2) or cardiovirus (3 amino acids within the last 15 amino acids are acidic, Table 2). These observations suggest that 2A proteases of enteroviruses but not other distinctly related picornaviruses (e.g. rhinoviruses, cardioviruses) possess transcriptional activity. Interestingly, a previous report demonstrated that this acidic region of poliovirus 2Apro is critical for viral RNA replication but not for cis- or trans- proteolytic cleavage [14]. Our results also demonstrated that EV71 2A protease without amino acids 146-149 still retained its protease activity (Figure 5A) but lost its transcriptional activity (Figure 5B). Thus, enteroviral 2A proteases may transactivate some cellular genes to benefit virus replication. Some cellular genes, e.g. PCBP2, PTBP1, RTN3, GBF1, CD55, and SAM68 gene, were reported to enhance the replication of poliovirus or EV71. However, EV71 2A protease suppressed rather than increased the transcription of these cellular genes (data not shown). These results were consistent with several reports regarding the shut-off of host cell mRNA synthesis caused by EV71 3C protein [32,33]. If enteroviral 2A proteases could in deed transactivate some cellular genes to benefit virus replication, further investigations are needed to determine its cellular target(s) and DNA-binding activity. Alternatively, EV71 2A protease may only help its own viral RNA synthesis in cytoplasm, whose mechanism is similar to the cellular transcription, rather than transactivate cellular genes to benefit virus replication. Further studies are needed to elucidate the function of this protein.

**Table 2:** The C-terminal 15 amino acid residues of picornaviral 2A protease sequences

Virus Name	GI	Sequence
Enterovirus type 71	66967945	DVRDLLWLDDEAMEQ
Coxsackie virus B3	323419	DIRDLLWLEDDAMEQ
Coxsackie virus B5	59045	DVRDLLWLEDDAMEQ
Coxsackie virus A17	238015862	SDIRDLYAYEEEAME
Poliovirus 1	193245090	DIRDLYAYEEEAMEQ
Poliovirus 1	193245074	DIRDLYAYEEEAMEQ
Poliovirus 2	332890	DIRDLYAYEEEAMEQ
Poliovirus	332895	DIRDLYAYEEEAMEQ
Poliovirus 3	61112	DIRDLYAYEEEAMEQ
Human rhinovirus 24	217316510	VAFIDLRHFHCADEQ
Human rhinovirus 52	217316506	CFADIRQLDFIAETQ
Human rhinovirus 94	217316500	VAFIDLRHFHCAEEQ
Human rhinovirus C	255115692	AFIDLRNYSSLSEHQ
Encephalomyocarditis virus	9626692	YFADLLIHDIETNPG

## Conclusions

In summary, 2Apro of enterovirus type 71 and Coxsackie virus B3 possesses transcriptional activity. The transcriptional activity of 2A protease was independent of its protease activity. Furthermore, the acidic domain at the C-terminus of 2Apro is essential for its transcriptional activity. Enteroviral 2A proteases may transactivate some cellular genes to benefit virus replication.

## Competing interests

The authors declare that they have no competing interests.

## Authors' contributions

CHY conducted majority of the experiments, HCL analyzed the data and wrote the manuscript, JGJ constructed the plasmids for Figures 1 and 4, CFH conducted the experiment of Figure 1, YJW conducted the experiment of Figure 3, MJL conducted the work of Figure 2, YLJ helped with the yeast two-hybrid experiment, and SYL designed the experiments and wrote the manuscript. All authors read and approved the final manuscript.
